# Cost analysis and provider preferences of low-dose, high-frequency approach to in-service training programs in Uganda

**DOI:** 10.7189/jogh.09.010416

**Published:** 2019-06

**Authors:** Michelle Willcox, Amnesty LeFevre, Enid Mwebaza, Josephine Nabukeera, Gabrielle Conecker, Peter Johnson

**Affiliations:** 1 Department of International Health, Johns Hopkins Bloomberg School of Public Health, Baltimore, Maryland, USA; 2 University of Cape Town, School of Public Health and Family Medicine, Division of Epidemiology and Biostatistics, Cape Town, South Africa; 3 Jhpiego, an affiliate of Johns Hopkins, Uganda; 4 Jhpiego, an affiliate of Johns Hopkins, Baltimore, Maryland, USA

## Abstract

**Background:**

Many countries in sub-Saharan Africa still face significant challenges in maternal and child health where low numbers, uneven distribution, and training deficits of the health workforce impede quality care. Low-dose, high-frequency training (LDHF), an innovative approach to in-service training, focuses on competency, team-based repetitive learning and practice in the clinical setting. In Uganda, we conducted cost analyses of local organization LDHF training programs for Post-abortion care (PAC) and Pediatric HIV to assess cost drivers and cost efficiency and compare costs to traditional workshop based training.

**Methods:**

We collected costs with bottom up, activity based costing in LDHF and workshop training programs. All costs reported from a programmatic perspective in US$2015 across a two year analytic time horizon. A survey of trained providers was conducted to understand costs and incentives of participation as well as experience and training preferences.

**Findings:**

PAC training with the LDHF approach cost US$29 957 corresponding to US$936 per provider; the traditional training of the same content was delivered at a total US$10 551 corresponding to US$527 per provider. Pediatric HIV training with LDHF approach cost US$41 677 or US$631 per provider; traditional training of Pediatric HIV cost US$18 656 or US$888 per provider trained. In traditional training programs, costs to providers were nearly equal to incentives given. In LDHF training programs, financial incentives and costs to participate were not equal and varied by roles and programs; all district trainers’ incentives outweighed their costs of participation, trainee incentives were higher than costs of participation in the PAC training, but in the Pediatric HIV program, trainee incentives were lower than the costs of participation.

**Conclusions:**

Local training programs differ widely in applying LDHF principles to design and implementation thus leading to variation in costs and cost-efficiency. LDHF can be more cost-efficient than workshop based trainings if programs take advantage of the wider scope of trainees available for the facility-based trainings. Incentive differences between district trainers and trainees may influence participation and perception of training. The perspectives of providers participating in LDHF or traditional workshop training should be integrated when developing future programs for maximum uptake and participation for in-service training.

Globally, 66% of maternal deaths and 50% of deaths in children under the age of 5 occurred in sub-Saharan Africa in 2015, the region has seen the least improvement in maternal, newborn, and child health (MNCH) over the last two decades [1-3]. Increasing coverage of key interventions and achieving the associated improvements in outcomes for MNCH requires significant human resources; however, often there is a lack of adequately trained health care providers where they are needed most [4,5]. Uganda faces public sector workforce shortages and slow growth with only 6.3 health professionals per 10 000 people available in 2015 (far below the WHO-recommended 25 per 10 000) [6,7]. Inadequacies in number and distribution of health providers combine with deficits in pre-service and in-service training to become a complex barrier to improving MNCH through the delivery of quality clinical care.

In-service training of health care professionals is a key strategy for improving the knowledge and capacity of existing health professionals to deliver high-quality care [8]. The low-dose, high-frequency (LDHF) capacity-building approach for in-service training delivers competency-focused content in small quantities with frequent repetition over time while promoting team-based, simulation-based practice with immediate feedback, and supervised direct patient care [9]. LDHF emphasizes on-site, facility-based training/simulation and follow-up where clinical practice occurs, minimizing disruption to service delivery while expanding the number of providers available to participate in the training and teambuilding. While evidence on LDHF training is limited [10,11], this approach may achieve similar or increased competency to previous classroom-based traditional workshop approaches while also reducing direct costs (travel refunds, per diems, hotel accommodation, and hall rental) and indirect costs (opportunity costs of leaving clinical practice and provider travel time to training).

With support from the Barr Foundation, Jhpiego-Uganda collaborated with the Association of Obstetricians and Gynecologists of Uganda (AOGU) and the Infectious Diseases Institute (IDI) of Makerere University to apply the LDHF approach to post-abortion care (PAC) and pediatric HIV/AIDS management, respectively. Both organizations conducted a comparison training utilizing a traditional workshop approach. The primary study objective was to explore the financial costs and cost-efficiency, or the cost per unit of provider trained, of utilizing the LDHF approach vs a workshop based training approach with each curriculum. The secondary objective was to examine provider preferences and perceptions of trainings through structured interviews with trainees from both deployments.

## METHODS

### Study setting

LDHF training for **PAC** was implemented in 16 health facilities (Health Center IIIs, IVs, and Hospitals) in the semi-urban districts of Wakiso and Mukono in the Central Region. Traditional PAC training included trainees from 18 health facilities in another Central Region district, Mpigi. In Uganda, illegal and often unsafe abortions contribute both to the need and complexity of PAC delivery [12]. As these PAC complications contribute significantly to maternal deaths, training has been recommended for high and midlevel health workers [13,14]. PAC is restricted further by absenteeism and task-shifting, which often leaves midwives stretched to provide services [15].

LDHF training for **Pediatric HIV/AIDS** management was implemented in 8 health facilities across four districts: Hoima and Kibaale in Western Uganda and Kyankwanzi and Kiboga in Central Uganda. Traditional training on pediatric HIV/AIDS was delivered for a separate group of providers representing 7 other facilities within the districts. Uganda’s high HIV general population rate at 7.4% in 2012 has been the focus of significant response resources, and the most recent National Strategic Plan outlines additional steps for prevention of mother to child transmission, provision of antiretroviral therapy (ART), strategies for early detection, treatment initiation, and management of ART especially for child cases of HIV [16-19]. Failures in early diagnosis and care linkages led to 72% of children presenting, in a 2012 study, with advanced disease at the initial visit to an HIV clinic and only 41% of eligible children receiving ART in 2013 [16,20]. The Ministry of Health has specified efforts to combat Pediatric HIV with communication strategies, community mobilization, and training for health care workers [21].

### Program description

The **PAC** program began in May 2015 with a two-month development phase. Start-up activities lasted one-month, which included District Health Team meetings, facility visits to select training sites, training of trainers (TOT) for selected district trainers, orientation and PAC content introduction for trainees, and baseline assessment data collection. Implementation spanned four months, during which the 12 district trainers led LDHF capacity-building for the 20 trainees – one-hour continuing medical education (CME) and one-hour hands-on practice – twice monthly until 6 training modules had been successfully completed. Final evaluation of trainees included a structured written knowledge assessment proctored by AOGU staff as well as an Observed Structured Clinical Exam (OSCEs) with five simulated case stations which tested providers’ skills.

**Pediatric HIV/AIDS** care LDHF training program began March 2015 with a two-month development phase including orientation meetings with the District Health Teams and facilities, baseline assessment, and LDHF curriculum development. Start-up activities included a two-week TOT for district mentors. The implementation phase included biweekly district mentor led LDHF sessions - CME trainings for 1 hour for 4 months (8 visits) with 2 months accounting for planning, delays, and follow up -open to all staff but targeted to 54 mentees. An additional 5 mentoring sessions were scheduled for district mentors to see patients with mentees, practice application of knowledge and provide feedback. The LDHF training targeted all cadres of staff involved in pediatric HIV/AIDS care services. The endline assessment included a written knowledge test and direct observation of skills during patient care visit using a scored checklist. **Table 1** defines the differences in the training programs, **Table 2** details activities differences of the LDHF training approaches, and **Table 3** summarizes which activities occurred between the programs’ different LDHF and traditional training approaches.

**Table 1:** Low dose, high frequency (LDHF) program descriptions

	PAC	Pediatric HIV
Program design	2 districts	4 districts
District TOT	6 days	10 days
Orientation/Initial training	2 days in Kampala	At facility visit
DHF training visits	6 visits (2 × per month, 3 months)	8 visits (2 × per month, 4 months)
District trainers	12	12
Providers trained	20	54
Number of health facilities	16	8
Selection criteria	Key health centers that frequently refer patients with post-abortion complications	Targeted health centers with multiple cadres responsible for all components of HIV care
Knowledge and competency assessments	True/False written assessment; Observed structured clinical exam (OSCE) with 5 stations	Multiple choice and True/False written assessment; Direct observation of clinical practice with key skills checklist

LDHF – low dose, high frequency, TOT – training of trainers, PAC – post-abortion care

**Table 2:** Training program by phase: development, start up, implementation activity descriptions for low dose, high frequency (LDHF) training approaches

	LDHF training for PAC		LDHF training for Pediatric HIV	
**Activity**	**Description**	**Time**	**Description**	**Time**
**Development:**				
Curriculum adaptation	Develop training manual	Mar	Adapt facility based curriculum from workshop and other existing materials	May
Initial stakeholder meeting	1 d meeting, 39 attendees from AOGU, health facilities, MOH, Jhpiego	Jun	n/a	
Baseline facility visit	Facility visit by 3 person team to assess equipment, services, and training needs, 3 d per district	May	n/a	
Personnel	AOGU staff with effort on LDHF	3 months	IDI staff with effort on LDHF	1 month
Furniture and Equipment	Allocated portion of AOGU Furniture and equipment	3 months	Allocated portion of furniture/equipment	1 month
Office space, utilities	Allocated building maintenance, utilities cost	3 months	Allocated building maintenance, utilities	1 month
**Start-up:**				
Orientation	District mentor (TOT) & Trainee Orientation: 2 d district mentor (12 district mentors) meeting, followed immediately by 2 d trainee (20 trainees) orientation	Jul	Facility visits to orient participants on LDHF	Jun
District trainer training (TOT)	District Mentor ward training: 2 d additional hands-on training for district mentors at Mulago hospital	Jul	District mentors: 2 week training of trainers for 12 district mentors in Kampala	Jun
Medical supplies	Post abortion care supplies and equipment	Jul	n/a	
Personnel	AOGU staff with effort on LDHF	1 month	IDI staff with effort on LDHF	2 month
Furniture and Equipment	Allocated portion of AOGU Furniture and equipment cost	1 month	Allocated portion Furniture and equipment cost	2 month
Office space, utilities	Allocated building maintenance, utilities cost	1 month	Allocated building maintenance, utilities cost	2 month
**Implementation:**				
Training delivery	LDHF facility training visits: District mentors visit 2 × per month, 3 months	Jul-Sept	LDHF facility training sessions: Visits by district mentors, lunch allowance for providers	Jul-Sept
Support supervision	4 visits by AOGU 3 person team	Aug-Sept	4 visits made over course of LDHF trainings by IDI team (2 persons)	Jul-Sept
Monitoring & evaluation	Endline Facility Visit: Facility visit by 1 person to assess equipment and services at end of program	Nov	2 M&E visits by IDI team to observe LDHF training session and follow up with participants	Jul-Sept
Endline provider evaluation	Mentee Evaluation Meeting: LDHF participant cost of total 1 d meeting, 52 attendees (AOGU staff, LDHF participants, traditional training participants)	Dec	Endline LDHF: Final assessment visit to facility by IDI team	Nov
Final stakeholder meeting	1 d meeting, 40 attendees: AOGU, MOH, training participants, Mulago hospital stakeholders, Jhpiego	Dec	n/a	
Personnel	AOGU staff with effort on LDHF	5 months	IDI staff with effort on LDHF	6 months
*Furniture and Equipment*	Allocated portion of AOGU Furniture and equipment cost	5 months	Allocated portion of AOGU Furniture and equipment cost	6 months
*Office space, utilities*	Allocated building maintenance, utilities	5 months	Allocated building maintenance, utilities	6 months

PAC – post-abortion care, TOT – training of trainers, AOGU – Association of Obstetricians and Gynecologists of Uganda, d – day

**Table 3:** Development, start-up, and implementation: differences in low dose, high frequency (LDHF) and traditional approach activities by phase

	Activity	Post abortion care		Pediatric HIV	
**LDHF**	**Traditional training**	**LDHF**	**Traditional training**
Development	Curriculum adaptation	×	–	×	–
	Initial stakeholder meeting	×	–	–	–
	Baseline facility visit	×	–	–	–
	Personnel	×	–	×	–
	Furniture and Equipment	×	–	×	–
	Office space, utilities	×	–	×	–
Start-up	Orientation	×	–	×	×
	District trainer training (TOT)	×	–	×	–
	Medical supplies	×	–	–	–
	Personnel	×	×	×	×
	Furniture and Equipment	×	×	×	×
	Office space, utilities	×	×	×	×
Implementation	Training delivery	×	×	×	×
	Support supervision	×	–	×	–
	Monitoring & evaluation	×	–	×	–
	Endline provider evaluation	×	×	×	×
	Final stakeholder meeting	×	–	–	–
	Personnel	×	×	×	×
	Furniture and equipment	×	×	×	×
	Office space, utilities	×	×	×	×

TOT – training of trainers

### Measurement

We prospectively monitored costs to determine full training program cost of the LDHF and traditional workshop approaches for each partner from March to December 2015. Costs were disaggregated into three distinct windows of time: development (two months), start-up (one month) and implementation (six months). Development and start-up activities were considered investments with an estimated useful life of 2 years and were amortized, or reduced and calculated to be used gradually over time, using a 3% discount rate; the analytic time horizon of 6 months reflects the implementation phase. Development and start-up activity costs included initial stakeholder meetings, adapting the curricula for LDHF, procuring medical supplies, holding the district trainer trainings and orientations, and costs of personnel. Implementation phase activities of the program included costs of personnel time, LDHF training sessions at facilities, evaluation of trainees as well as building and support costs. Furniture and equipment, which did not have a local cost data source, were valued by standards of the region set forth by WHO CHOICE [22]. Final costs presented in 2015 US$ for a 9-month analytic time horizon aligned with program implementation.

We additionally sought to assess opportunity costs of provider participation in trainings through a participating provider survey conducted as a quasi-independent study with Jhpiego Uganda and Johns Hopkins School of Public Health. The survey included quantitative questions on demographics and training participation and open-ended questions to elicit qualitative responses about preferences and perceptions of training.

### Sampling

**PAC** training with the LDHF approach was delivered to 20 health providers in 16 facilities while the traditional workshop approach was utilized to train a comparator group of 20 health providers from 18 facilities. Once identified as target health facilities for post-abortion complication referrals, the nurse, physician, or manager ‘in-charge’ of the facility selected one or more health providers to attend the training.

LDHF training in **Pediatric HIV/AIDS** management was delivered to 54 health professionals in 8 facilities, while 21 providers in 7 separate facilities were trained through a traditional workshop based approach. The implementing organization selected eligible staff with facility leadership recommendation; participants included clinical officers, registered nurses, laboratory technician/assistants, enrolled nurses, midwives, or records assistants that were responsible for clinical care and management of HIV/AIDS care.

The provider survey sampling sought 100% of training participants (139) from all roles of LDHF trainee, traditional trainee, and district mentors by visits of research assistants to health facilities and districts; scheduling issues and provider leave contributed to an 8% loss to follow up. Of the 119 providers surveyed (48 from PAC and 71 from Pediatric HIV), 58 were LDHF trainees, 20 were LDHF district mentors, and 39 were traditional trainees.

### Data collection

All training program activities and resources were defined through semi-structured interviews by external costing experts with personnel from each training program. Cost data were then collected by training program personnel using a standardized Excel tool developed externally based on the reported activities outlined through semi-structured interviews with program personnel. Cost data were sourced from financial cost reports, receipts, accounting systems, or other data sources identified by the training partner staff who had participated in program costing methods instruction.

The training participant provider survey was developed, pilot tested, and programmed a tablet-based, 45-minute to 1 hour in-depth interview conducted in English with local language clarifications by Ugandan midwives trained as research assistants in qualitative data. The survey collected key data on provider income, financial incentives of trainings, and time spent on and travel to either training. Open-ended responses were captured in handwritten form by the interviewer and entered digitally into the tablet after the interview concluded to ensure interviewers focused on responents rather than devices.

### Analysis

Cost analysis was conducted by costing experts Johns Hopkins Bloomberg School of Public Health in MS Excel (Microsoft Inc, Seattle WA, USA) to summarize and evaluate cost drivers and activity resource allocation for each implementing partner program. Cost analyses included assessing the costs and cost-efficiency of conducting in-service training through LDHF approach and traditional workshop approaches in both PAC and Pediatric HIV training programs. Provider survey data were collected in the Mobile Data Studio platform, transferred to excel, and analyzed in Stata Version 13.1 (Stata Corp, College Station TX, USA). Data from the open-ended provider surveys were analyzed following general qualitative thematic analysis guidelines. Responses were manually coded through an iterative process; themes and sub-themes were identified and responses were re-analyzed in order to code presence of these defined concepts in each response.

### Ethics approval

The institutional review board (IRB) at the Johns Hopkins University, Bloomberg School of Public Health in Baltimore, Maryland, USA determined the study protocol to be non-human subjects research. This IRB determination was shared with local partners and each conducted their own ethics review process as well.

## RESULTS

### Costs of in-service training for PAC

PAC LDHF training had an estimated financial cost of US$52 680, including US$30 761 to develop and start-up program activities and US$21 919 to implement the LDHF training sessions (**Table 2**). Of total cost for the training program, one-time development and start-up main costs were medical supply procurement (16%), District Trainer TOT and Trainee Orientation (16%), curriculum adaption (8%), personnel (7%), and initial stakeholder meetings (4%). Of the total program costs, implementation costs were driven by LDHF facility based training (11%), personnel costs (8%), supportive supervision (5%), final stakeholder meetings (5%), monitoring and evaluation (4%), endline provider evaluation (4%), and other costs (**Table 4**). When amortizing development and start-up phase costs over the expected 2-year lifespan of the training, the total cost for the LDHF training was US$29 957 and the cost per person trained was US$936. For each training approach, details on total financial cost are found in **Table 4** and details on total annualized costs found in **Table 5**.

**Table 4:** Total financial costs for low dose, high frequency (LDHF) and traditional training programs

		Post abortion care				Pediatric HIV			
**Program costs**		**LDHF, 9 months**		**Traditional training, 1 month**		**LDHF, 9 months**		**Traditional training, 1 month**	
	**Activity**	**Cost (US$)**	**%**	**Cost (US$)**	**%**	**Cost (US$)**	**%**	**Cost (US$)**	**%**
Development	Curriculum adaptation	4386	8%			3639	6%		
	Initial stakeholder meeting	2350	4%						
	Baseline facility visit	1814	3%						
	Personnel	2674	5%			2805	4%		
	Furniture and Equipment	580	1%			213	0%		
	Office space, utilities	618	1%			606	1%		
Start-up	Medical supplies	8613	16%						
	Orientation	5571	11%			1670	3%	1038	5%
	District trainer training (TOT)	2863	5%			15 000	23%		
	Personnel	891	2%	244	2%	5611	9%	767	4%
	Furniture and Equipment	193	0%	48	0%	426	1%	147	1%
	Office space, utilities	206	0%	56	1%	1212	2%	166	1%
Implementation	Training delivery	5886	11%	8378	78%	5081	8%	13125	65%
	Personnel	4457	8%	731	7%	16 832	26%	2301	11%
	Support supervision	2537	5%			4396	7%		0%
	Monitoring & evaluation	2229	4%			291	0%		0%
	Endline provider evaluation	2364	4%	1149	11%	2016	3%	1737	9%
	Final stakeholder meeting	2450	5%				0%		0%
	Furniture and Equipment	967	2%	145	1%	1279	2%	442	2%
	Office space, utilities	1030	2%	56	1%	3635	6%	497	2%
Total program cost		**52** **680**		**10** **808**		**64** **712**		**20** **220**	
Total program cost per recipient of training		**1646**		**540**		**980**		**963**	

**Table 5:** Total annualized program costs for LDHF and traditional training programs

Activity		Post abortion care						Pediatric HIV					
		LDHF training			Traditional training			LDHF training			Traditional training		
Annualized 2 year cost (US$)*	**Amortized 6 month cost**	**%**	**Annualized cost (US$)**	**Amortized 6 month cost**	**%**	Annualized cost (US$)*	**Amortized 6 month cost**	**%**	**Annualized Cost (US$)**	**Amortized 6 month cost**	**%**
Development	Curriculum Adaptation	2292	**1146**	4				1902	**951**	2			
	Initial Stakeholder Meeting	1228	**614**	2									
	Baseline Facility Visit	948	**474**	2									
	Personnel	1398	**699**	2				1466	**733**	2			
	Furniture and Equipment	303	**152**	1				111	**56**	0			
	Office space, utilities	323	**161**	1				317	**158**	0			
Start-up	Orientation	2912	**1456**	5				873	**436**	1	543	**271**	1%
	District trainer training (TOT)	1496	**748**	2				7839	**3920**	9			
	Medical Supplies	4501	**2251**	8									
	Personnel	466	**233**	1	127	**64**	1	2932	**1466**	4	401	**200**	1%
	Furniture and Equipment	101	**51**	0	25	**13**	0	223	**111**	0	77	**38**	0%
	Office space, utilities	108	**54**	0	29	**15**	0	633	**317**	1	87	**43**	0%
Implementation	Training delivery	23543	**5886**	20	33513	**8378**	79	20322	**5081**	12	52500	**13125**	70%
	Support Supervision	10149	**2537**	8	0		0	17584	**4396**	11	0		
	Monitoring & Evaluation	8914	**2229**	7	0		0	1163	**291**	1	0		
	Endline Provider Evaluation	9455	**2364**	8	4596	**1149**	11	8062	**2016**	5	6948	**1737**	9%
	Final Stakeholder Meeting	9800	**2450**	8	0		0	0		0	0		0%
	Personnel	17 829	**4457**	15	2925	**731**	7	67 328	**16** **832**	40	9205	**2301**	12%
	Furniture and Equipment	3867	**967**	3	580	**145**	1	5116	**1279**	3	1767	**442**	2%
	Office space, utilities	4120	**1030**	3	225	**56**	1	14 541	**3635**	9	1988	**497**	3%
Total Annualized Cost		103 752	**29** **957**		42021	**10** **551**		150 413	**41** **677**		73 515	**18** **656**	
Total Participants		128	**32**		**80**	**20**		**264**	**66**		84	**21**	
Total cost per training recipient			**936**			**527**			**631**			**888**	

LDHF – low dose, high frequency, TOT – training the trainers

*2-year cost model assumptions: additional providers ready to be selected and participate in LDHF or Traditional training, no new district trainers trained, no new medical supplies purchased for additional trainings.

PAC workshop training corresponded to a total financial program cost of US$10 808, including $348 for one-time start-up activities and US$10 460 for implementation. The program had two main activity cost drivers; 78% of traditional training costs were incurred during the workshop (US$8378) and 11% of costs (US$1149) were incurred during a shared final day evaluation (**Table 4**). Personnel salary cost to support traditional training was low as staff had only a short time devoted to planning. Salaries were US$975 or 9% of the traditional training cost (**Table 4**). After amortizing the start-up activities over the expected 2-year lifespan of the training, the estimated cost for a 6-month implementation period for traditional training was US$10 551, with a corresponding cost per training recipient of US$527 (**Table 5**).

### Provider incentives and costs of participating for PAC

The cost to participate – travel, transport, and opportunity cost of income that could have been generated in the time spent on the training program – for the PAC LDHF training differed between district trainers and LDHF trainees. District trainers had a total cost to participate of $192 and were given a total facilitation incentive of US$376, including the 6-day training of trainers and 6 LDHF sessions conducted. The LDHF trainees had a total cost to participate of $49 and were given a total facilitation incentive (lunch, travel) of US$66. The facilitation incentives were given by the training organization and are included in the programmatic costs listed above; the cost to participate is drawn from the provider survey data for district trainers and LDHF trainees (**Table 6**).

**Table 6:** Demographics of provider survey respondents for PAC and Pediatric HIV

	PAC (N = 48)	Pediatric HIV(N = 71)
**Provider background:**		
Sex	10% M	42% M
	90% F	58% F
Age (mean, 95% confidence interval)	40 (25-57)	35 (22-60)
**Marital status:**		
Married	58.3%	66.2%
Single	35.4%	26.8%
Widowed/divorced	4.7%	5.6%
Other/Did not disclose	2.1%	1.4%
**Education:**		
Ordinary level	0%	11.6%
Advanced level	4.8%	1.7%
Certificate	23.8%	40.0%
Diploma	47.6%	43.3%
Degree	19.0%	3.3%
Postgraduate	4.8%	0%
**Designation/title:**		
Medical officer	2.1%	0%
Clinical officer	12.5%	12.7%
Enrolled nurse	0%	7.0%
Registered nurse	8.3%	11.3%
Comprehensive nurse	0%	1.4%
Enrolled midwife	45.8%	18.3%
Registered midwife	29.2%	1.4%
Laboratory assistant	0%	11.3%
Laboratory technician	0%	14.1%
Other (records assistant, student)	2.1%	22.5%
**Years of experience**	14.6	8.3
**Promoted since last professional course**	25%	14%
**Reason for promotion:**		
Years of service	33.3%	40%
Attendance of training	8.3%	0%
Attendance, facility based training	0%	10%
Other (upgrading qualifications, good performance, position vacancy)	58.3%	50%

PAC – post-abortion care

The provider cost to participate as a trainee in the PAC workshop training was a total of $38. Workshop trainees were given a total facilitation incentive (lunch, travel) of US$34. The facilitation costs were included in the programmatic costs listed above, whereas the cost to participate indicate the opportunity cost for traditional workshop trainees.

### Costs of in-service training for Pediatric HIV

Pediatric HIV/AIDS training through the 9-month LDHF program had a total financial cost of $64 712, including development and start-up activities with an estimated cost of US$31 183 (48% of overall program cost) and the cost of LDHF training session implementation which was US$33 529. The cost driver of one-time start-up activities was the two-week District Mentor TOT at US$15 000 – 23% of the overall program cost. Among recurrent costs, personnel salary and benefits were the leading drivers; accounting for 39% of the total program cost (**Table 4**). The provision of training itself was conducted through 8 visits by district mentors at a cost of US$5081 (8% of program cost) and 4 supportive supervision visits costing an estimated US$4396 (7% of program cost) (**Table 4**). When amortizing development and start-up phase costs over the expected 2-year lifespan of the training program, the 6-month implementation of the LDHF training cost an estimated US$41 677 and the cost per training recipient US$631 (**Table 5**).

Pediatric HIV/AIDS training through the traditional workshop corresponded to a total of $20 220 in financial costs over the course of 1 month, including $2118 used for start-up activities and $18 102 for implementation activities. Over half of the total program budget, US$13 125 (65%), was spent on the conduct of traditional training workshop. Another cost driver during the implementation phase was personnel (11%) and endline provider assessment (9%), which took place at the facility and was conducted by staff (**Table 4**). After annualizing development and start-up activities, the estimated cost for a 6-month implementation period for traditional training was US$18 656, with a corresponding cost per training recipient of US$888 (**Table 5**).

### Provider incentives and costs of participating in-service training for Pediatric HIV

The cost to participate – travel, transport, and opportunity cost of income that could have been generated in the time spent on the training program – for the Pediatric HIV LDHF training differed between district trainers and LDHF trainees. District trainers had a total cost to participate of US$220 and were given a total facilitation incentive of US$429, including a 10-day training of trainers and average 8 LDHF sessions conducted. The LDHF trainees had a total cost to participate of US$33 and were given a total facilitation incentive (lunch, travel) of US$17. The provider cost to participate as a trainee in the pediatric HIV workshop training was a total of US$36. Workshop trainees were given a total facilitation incentive (lunch, travel) of US$34 (**Table 7**).

**Table 7:** Incentives and costs to participate by training and participant type

Participants by role and training program	PAC			Pediatric HIV			Source
**LDHF training**		**Traditional workshop trainees**	**LDHF training**		**Traditional workshop, trainees**
**District trainers**	**LDHF trainees**		**District trainers**	**LDHF trainees**	
Number of participants	12	20	20	12	54	21	
Provider income: Daily income value from all income sources	US$19	U$10	US$9	US$11	US$8	US$7	Provider survey
Off-site Training	LDHF TOT	LDHF Orientation	Traditional Workshop	LDHF TOT	LDHF	Traditional Workshop	
Days spent in training (per provider)	5.1	2	4	14	no off-site	5	Provider survey, Program data
A. Provider cost to participate: Value of provider time (duration×daily income value)	**US$97**	**US$19**	**US$38**	**US$157**		**US$36**	Provider survey
B. Program incentive to providers: Total (US$) given to participants for offsite training	**US$74**	**US$14**	**US$34**	**US$286**		**US$34**	Program costs, provider survey
On-site training	LDHF Sessions	LDHF Sessions	**no on-site**	LDHF Sessions	LDHF Sessions	**no on-site**	
Total days spent (hours per session×Number of sessions)	3.0	1.7		3.6	2.6		Provider survey
Value of provider time spent during training (duration of LDHF×sessions completed×daily income value)	US$56	US$17		US$41	US$21		Provider survey
Value of travel for all LDHF sessions (time value and transport cost, US$)	US$39	US$12		US$22	US$12		Provider survey
C. Provider cost to participate: Total cost of time and travel for On-site LDHF sessions	**US$95**	**US$29**		**US$62**	**US$33**		Provider survey
D. Program incentive to providers: Incentive total given for LDHF sessions completed	**US$302**	**US$51**		**US$143**	**US$17**		Program costs, provider survey
Total provider cost to participate (A+C)	**US$192**	**US$49**	**US$38**	**US$220**	**US$33**	**US$36**	
Total incentive from organization for training program participation (B+D)	**US$376**	**US$66**	**US$34**	**US$429**	**US$17**	**US$34**	
Difference between incentive received and provider cost to participate	US$184	US$17	-US$4	US$209	-US$16	-US$2	

LDHF – low dose, high frequency, PAC – post-abortion care

### Provider preferences for LDHF training

**Figure 1:**
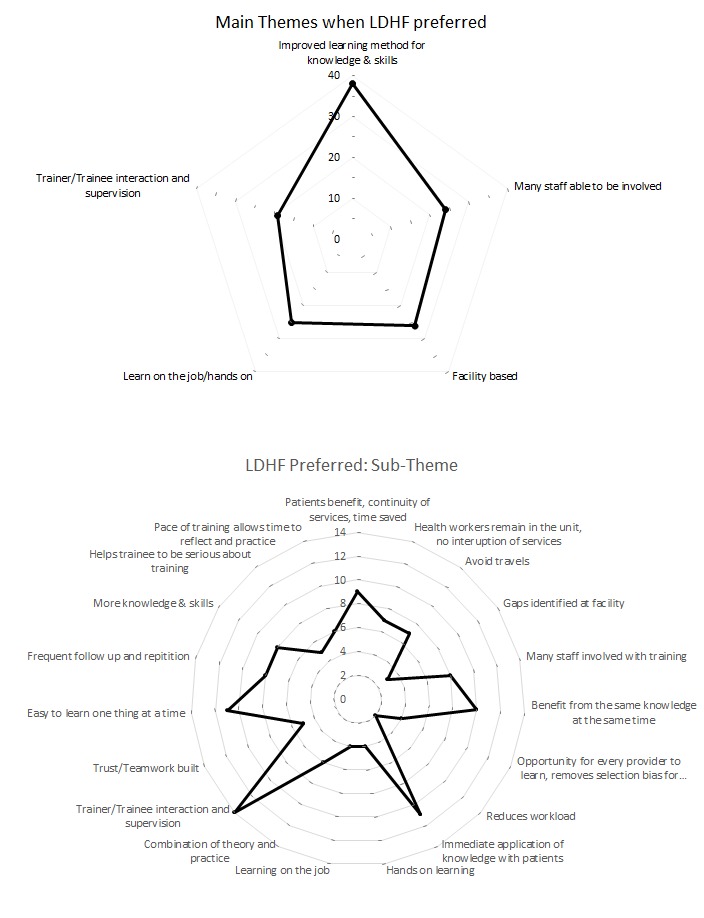
Thematic preferences among providers that prefer low dose, high frequency (LDHF) over traditional training.

The responses by training program participants provided insight on provider preferences and perspectives regarding LDHF training and traditional workshop training. Among those who had direct experience with the LDHF approach, 70% reported preferring LDHF as compared to traditional training. Most providers that preferred LDHF felt that it improved learning methods for transferring knowledge and critical skills, provided a forum for including and engaging many staff, and was beneficial for its facility location, allowance of hands-on learning, and interaction with supervisors for the mix of theory and practical experience (**Figure 1**).

Providers felt LDHF facilitated course focus in small manageable quantities and accommodated practice.

*I prefer LDHF because we grasp little by little making it easy to learn, and because of the frequency, remembering is easy*. – LDHF mentee, Pediatric HIV, Nursing student

Respondents noted greater transfer of knowledge and skills among providers in the facility and helpfulness of frequent follow-up and repetition.

*LDHF is preferred to traditional methodology, because some of the health care workers who attend the traditional trainings fail to understand the importance of passing on the same knowledge to the colleagues*. – LDHF mentee for Pediatric HIV, Lab Technician

Whereas traditional trainings often select only one or two providers from a health facility, LDHF was perceived to benefit more participants and promote teamwork at the facility. Another location-based benefit relayed by respondents noted that LDHF training on-site saved patients’ time, avoided service provision interruptions, minimized provider absenteeism, and gave hands-on learning opportunities with outside facilitators.

*I prefer LDHF because in terms of knowledge attainment it gives a high percentage. It saves time because it is done at the facility. Patients benefit because health workers are at the facility*. – LDHF mentee for Pediatric HIV, Clinical Officer

Holding the training at the facility saved travel time and allowed more time to apply concepts to patient care.

*Prefer LDHF because trainers find you at the facility. No transport incurred as in traditional [workshop training] which you have to travel to the training sight [sic]. You practice on already available patients which is not the case in traditional workshops where we learn on dolls*. – LDHF mentee, Pediatric HIV, Lab Assistant

Facility-based LDHF sessions were seen to provide opportunities for interaction and adequate time to address an individual’s learning needs or provide immediate feedback.

*LDHF is better, you have enough time to sit down with the mentees so that you understand better the individual needs. It gives more time to the mentees to practice on the tools, clients and the Laboratory investigation*. – LDHF district mentor, Pediatric HIV, Medical Records Assistant

Respondents emphasized that LDHF provided a better balance of theory and patient-based application of skills in the clinical setting than traditional workshops.

*This LDHF training…because of the combination of practice and the theoretical part of it. We had the problem of filling the EID clinical care cards and the register. During the training, they understood better since they filled the card together with the mentor then transferred the information to the register directly and immediately*. – LDHF mentee, Pediatric HIV, Enrolled Midwife

### Provider preferences for traditional workshop trainings

**Figure 2:**
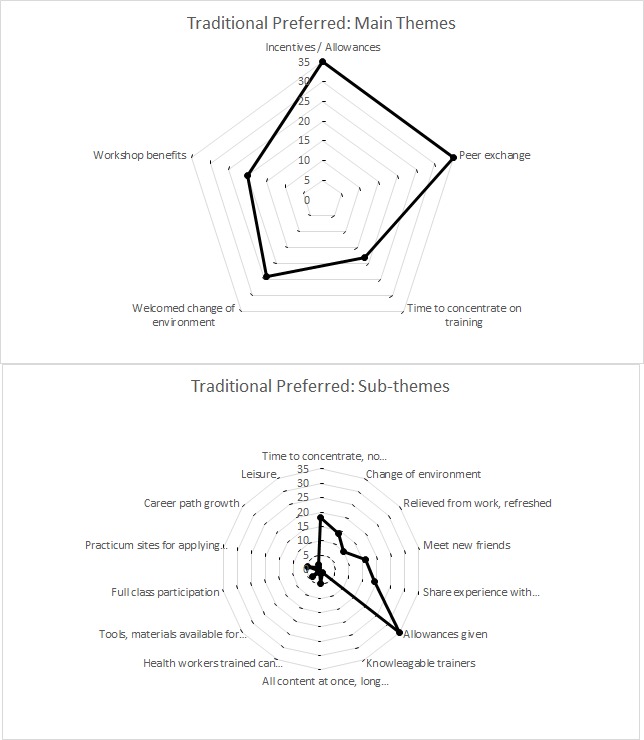
Thematic preferences among providers that prefer traditional training over low dose, high frequency (LDHF).

Despite the many advantages of LDHF, 45% of all survey respondents favored traditional training. Primary reasons included uninterrupted time to concentrate during a workshop, refreshing change of environment, valuable peer exchange, better incentives, and access to new resources with centralized workshop structure (**Figure 2**).

Many preferred traditional training off-site due to time and concentration away from patient interruptions.

*Workshop[s] are better especially those residential because the[re] would be no disturbance just focusing on training unlike on-site where you are called on and off*. – LDHF mentee, Pediatric HIV, Records Assistant

They also noted that a brief reprieve from work and chance to leave the facility was a refreshing change.

*Traditional because it enables you to change the environment and makes you feel like you have rested as if you have been on leave*. – Traditional training participant, PAC, Clinical Officer

The chance to meet, interact, build friendships and network professionally was a key theme among respondents who preferred traditional workshop training. Shared experience with new colleagues was highly valued.

*Traditional because we share experience with the other colleagues from the different types of health settings, that is learning what one needs…and this helps us to lobby for what we don't have in our facilities*. – LDHF mentee, Pediatric HIV, Lab Technician

Respondents cited the higher incentives of the traditional training were preferred to those of the LDHF approach.

Providers noted other traditional workshop structure benefits: specialists and multiple trainers, new equipment accessible in central locations, and easier to maintain participation and convey all content at once.

## DISCUSSION

Low-dose, high-frequency training principles emphasize competency-focused content, simulation and case-based learning, appropriately spaced and brief periods of training, in-service learning, team approach and peer leadership, and supported ongoing practice [9]. In Uganda, implementing partners participated in the initial Jhpiego-led workshop on the LDHF approach and the same overall budget for the training program. However, each program evolved and established unique LDHF delivery strategies, personnel teams, district trainer preparations, numbers and cadres of participants, assessments and incentives. These differences contributed to variation in cost drivers and overall cost-efficiency of reaching a provider and improving critical competencies.

In exploring barriers to accepting LDHF, we hypothesized that providers might resist the transition from a traditional workshop to the LDHF approach for training delivery given differences in financial incentives as training per diems or allowances have been considered a supplement to low wages [23,24]. We estimated the opportunity cost to providers by measuring the daily income value from all income-generating activities as well as value of time spent in LDHF as compared to traditional training. This cost to participate in the traditional training was similar for both PAC and Pediatric HIV, US$38 and US$36, respectively. This is slightly higher than the actual incentive given to traditional training participants; thus, on average, traditional workshop trainees could in fact have earned slightly more if they had forgone the workshop and opted to continue with their other income-generating activities – US$4 among PAC group and US$2 among Pediatric HIV group. This finding is contrary to popular opinion that off-site training incentives are excessive and appealing because so much higher than normal income.

In LDHF training, shorter duration and facility-based follow-up translated to lower overall incentives received by trainees. In comparison to the traditional workshop trainee incentives, the incentives for LDHF trainees did not include per diem, accommodation, or transport. Between the LDHF approaches, incentives varied significantly depending on organization and role as trainer or trainee. Over the course of the PAC training, LDHF trainers received US$376, the LDHF trainees received US$66 on average. The Pediatric HIV program also had similar variation in total incentives; LDHF trainers received US$429, LDHF trainees received US$17 which did not cover their estimated costs of participation. These variations may address unique aspects of the LDHF programs such as travel difficulty, time requirements, or participant skill levels; however, standardization of participants’ incentives based on time estimated for participation should be considered in future LDHF programs. After accounting for the cost to participate, PAC trainees earned more with incentives provided by the LDHF program than their cost to participate and more than they would have earned from participating in the traditional workshop. Pediatric HIV trainees experienced the opposite; the US$17 LDHF program incentives did not cover the average US$33 costs to participate which made them worse off than they would have been without the program and at a significant opportunity cost from their alternative participation in the traditional workshop.

Providers who preferred LDHF believed this approach at the facility led to better learning, helped patient care, engaged many staff, and allowed hands-on learning with supervisor interaction. Respondents’ perspectives on potential barriers to acceptance for the LDHF approach shared key themes related to concern for increased patient wait times and workload, low staffing and scheduling issues, low attendance of all sessions, and small financial incentives. Future training program design, should closely consider these preferences and perspectives. If trainees do not accept and engage in the training, lack of participation could undermine potential improvements in competency.

### Comparison with other training programs

Continuing medical education can be very effective in capacity building, however the many moderating variables and poor quality of cost-effectiveness studies which assess costs and measured outcomes for multiple training alternatives have inhibited the development of clear and concise guidelines on the optimal training delivery methods for different cadres and practicing sites [25-28]. Comparisons across the processes and structures of different LDHF training programs and traditional training alternatives has led to a more robust understanding of the feasibility of large-scale LDHF programs. LDHF training principles have been incorporated with promising results for maternal and newborn health in Uganda and Ghana as well as in high-income country hospitals for a training dose-response in higher retention of CPR skills with completion of additional on-site trainings [10,11,29]. Additionally, the literature suggests that training environments that are similar to those in which health providers practice are most efficient for the uptake of knowledge and skills and interactive, repetitive trainings improve learning as compared to didactic, singular trainings [30]. These benefits of LDHF were noted by providers in their perspectives of the approaches, but without acknowledging and accounting for provider preferences for peer networking, appropriate incentives, and chances to access expertise and new equipment these benefits may not be fully realized in future training programs.

### Limitations of study

The PAC program provider assessments could not be used to measure attributable effect of LDHF training on knowledge and competency due to changes in the written exam, modification of competency testing methods, and different providers selected to assess at baseline vs endline. The absence of this robust training effectiveness data and the short window of program implementation pose limitations for our study. Additional challenges during LDHF on-site training – for both the PAC and Pediatric HIV programs – such as absenteeism or human resource transfers affected attendance of training sessions. On average, PAC training participants attended 73% of LDHF sessions and pediatric HIV participants attended 60% of LDHF sessions, which may have affected knowledge and competency. Training organizations did collect some facility health service delivery data, but none of these data were used to measure the effectiveness of the training program due to the short window of implementation and challenges with attribution. Beyond challenges with program evaluation, funding and program delays influenced the work of all partners and led to vastly differing implementation and assessment timeframes for the LDHF program and the comparison traditional workshop. Future research studies of training approaches should adopt a longer analytic time horizon, evaluate a possible moderating effect of health worker turnover or absenteeism on knowledge and competency gains, and optimally measure value for money along with changes in service delivery and related health outcomes such as deaths or infections averted.

## CONCLUSION

Interpretation of the low-dose, high-frequency training as a concept varies greatly depending on the content, context, and implementing organization. The cost-efficiency of LDHF compared to traditional training depends on how many providers are reached and how the training is deployed. For the Pediatric HIV training, program costs per participant were lower in the LDHF approach than in the traditional approach, but the reverse was seen with the PAC training programs. The unique application of LDHF with two curricula and two different organizations has created an opportunity for collaboration and greater knowledge gained about this emerging training approach. The LDHF approach appeals to health providers and other stakeholders, but some preferences for workshop training persist. Future training programs need to understand the balance between costs of participation and incentives or training structures that encourage full participation and completion of LDHF training sessions; analyzing cost-effectiveness of these future programs will require connecting the knowledge and competency taught by the different approaches to changes in provider practice and patient health outcomes. LDHF training has potential as a cost-efficient approach to build capacity of health providers and, ultimately, improve patient care.
